# Obituary

**Published:** 1856-05

**Authors:** 


*Obituary.*—We are pained to learn the death of Dr. Henry Clark, an American Surgeon in the Russian Service, in the Crimea. Dr. Clark was the son (we believe the only one) of Dr. Henry Clark, of Livonia, Livingston County, N. Y., one of the oldest and most worthy practitioners in that section.

Tired of the inaction of a country life, thoroughly educated and full of ambition, Dr. Clark sought this opening to success, only to perish of typhus. Our acquaintance with him was only of a day's duration, but during that brief time, his manly qualities, high sense of honor, and thorough scholarship, made a lasting impression on our memory. We learn his death with sincere regret, and tender our earnest sympathies to his afflicted friends.

